# Supplementary figures and images for: Studying genetic population structure to shed light on the demographic explosion of the rare species Barbitistes vicetinus (Orthoptera, Tettigoniidae)
Source: PLoS One. 2021 May 6;16(5):e0250507. doi: 10.1371/journal.pone.0250507 (PMC8101909; doi:10.1371/journal.pone.0250507)

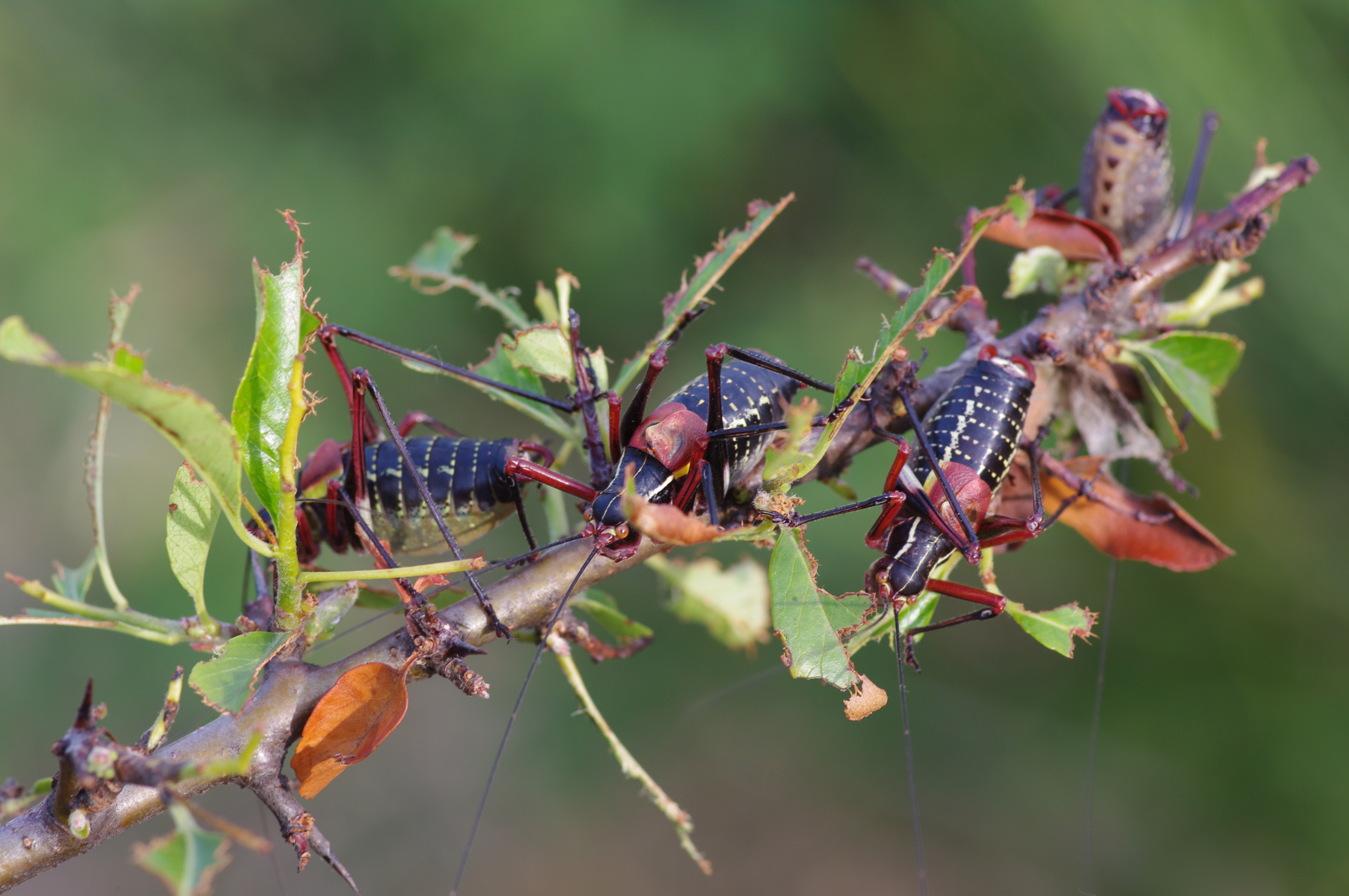

Supplement: S1 Fig — (TIF) [file pone.0250507.s001.tif]

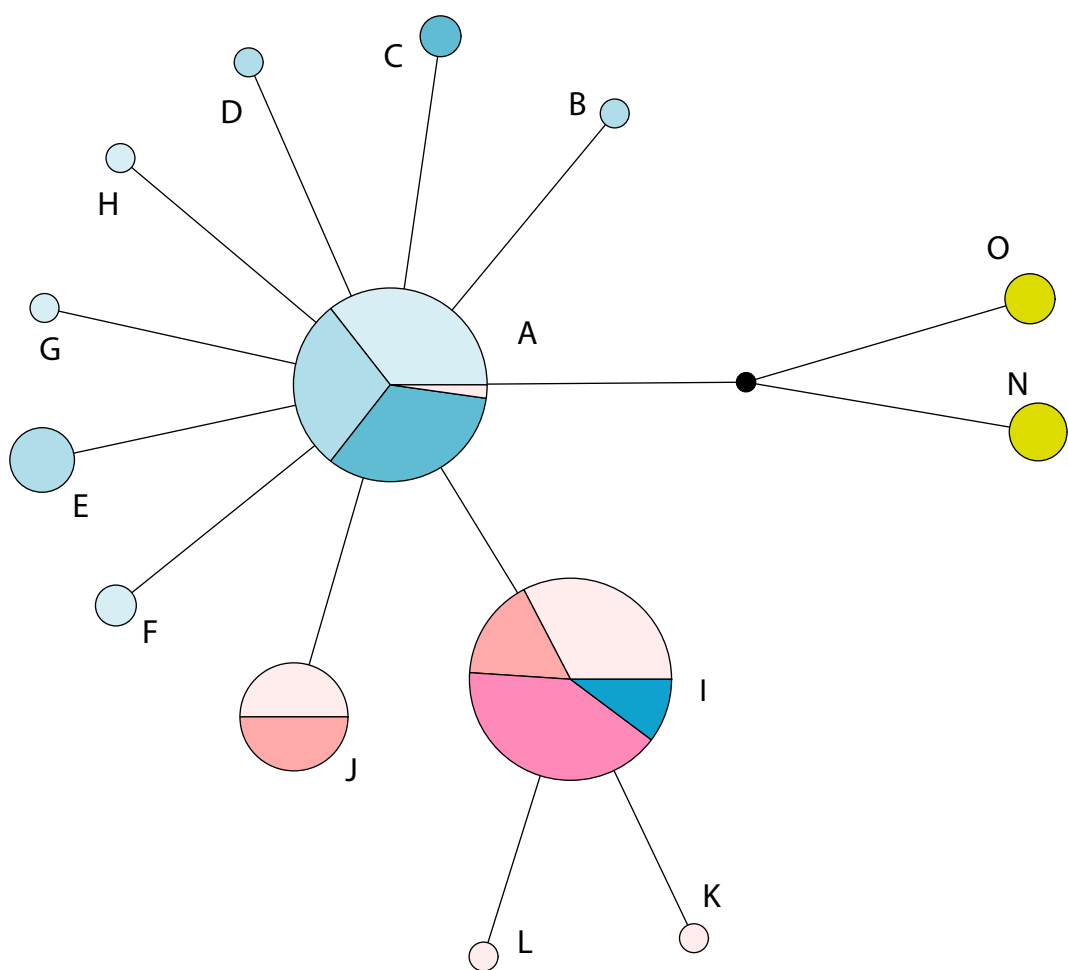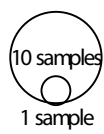

Euganean Hills

- North
- Center
- South

Berici Hills

- North
- Center
- South

Lessini Mountains

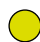

Supplement: S2 Fig — Network obtained with the COI-tRNALeu-COII data set. (PDF) [file pone.0250507.s002.pdf]

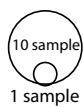

○ North  
● Center  
● South

○ North  
● Center  
● South

Supplement: S3 Fig — Network obtained with the COI-tRNALeu-COII data set. B) Network obtained with the 12S-CR data set. Network obtained with the 12S-CR data set. (PDF) [file pone.0250507.s003.pdf]

### Euganean Hills

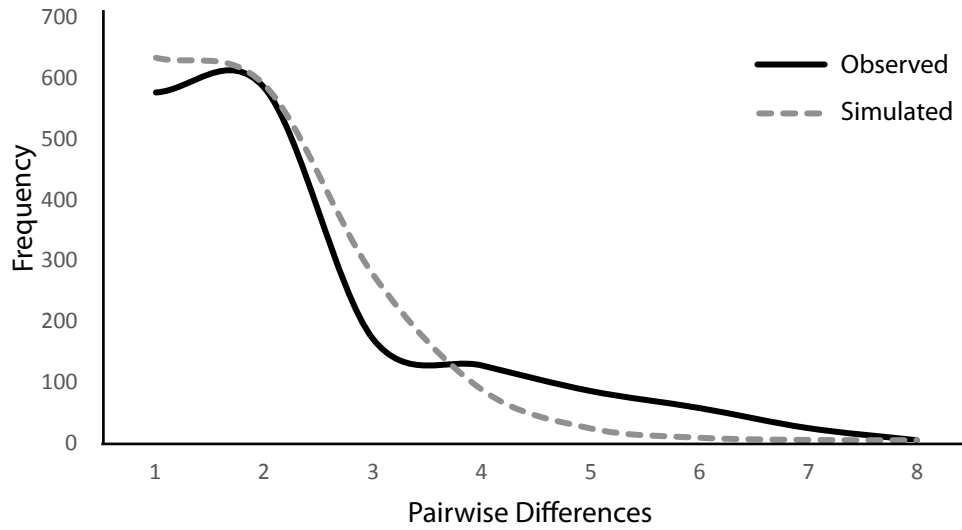

### Berici Hills

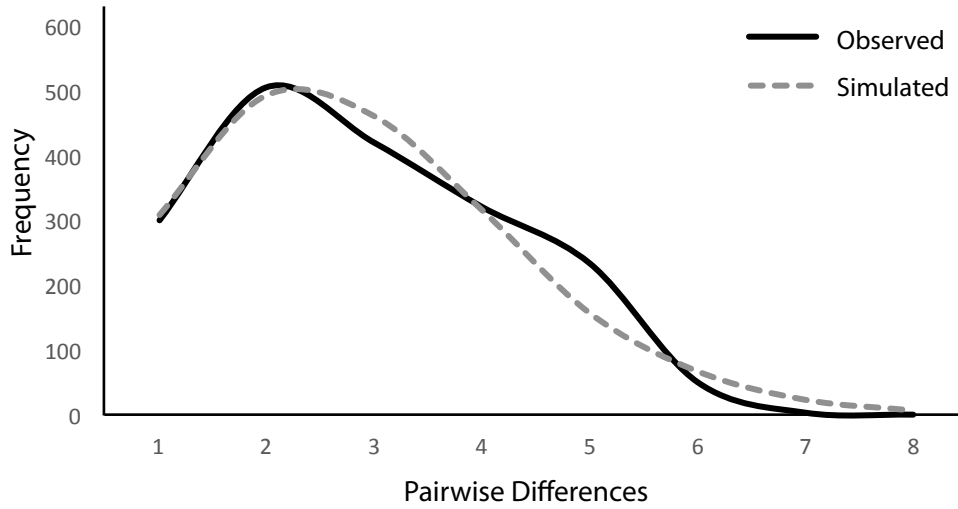

Supplement: S4 Fig — (PDF) [file pone.0250507.s004.pdf]
